# Increased Resting-State Functional Connectivity of the Hippocampus in Rats With Sepsis-Associated Encephalopathy

**DOI:** 10.3389/fnins.2022.894720

**Published:** 2022-06-02

**Authors:** Yue Yao, Chunqiang Lu, Jiu Chen, Jie Sun, Cuihua Zhou, Cheng Tan, Xian Xian, Jianhua Tong, Hao Yao

**Affiliations:** ^1^Cardiovascular Surgery Center, The Second Affiliated Hospital of Nanjing Medical University, Nanjing, China; ^2^Department of Radiology, Zhongda Hospital, Medical School of Southeast University, Nanjing, China; ^3^Institute of Brain Functional Imaging, The Affiliated Brain Hospital of Nanjing Medical University, Nanjing, China; ^4^Department of Anesthesiology, Zhongda Hospital, Medical School of Southeast University, Nanjing, China; ^5^Department of Anesthesiology, The Second Affiliated Hospital of Nanjing Medical University, Nanjing, China

**Keywords:** sepsis-associated encephalopathy, hippocampus, functional magnetic resonance imaging, functional network, functional connectivity

## Abstract

**Background:**

Sepsis-associated encephalopathy (SAE) has been identified as a frequent complication of sepsis, featured by an aberrant level of cognitive and affective functions. The present study is designed to explore the changes in functional connectivity (FC) of the hippocampus in rats with SAE utilizing resting-state functional magnetic resonance imaging (rs-fMRI).

**Methods:**

Sprague-Dawley rats were randomly assigned to the SAE and control groups. We acquired rs-fMRI data using a 7T MRI to evaluate hippocampal network functional differences between the two groups with a seed-based approach. Behavioral performance was assessed using the open field test and forced swimming test. Statistical analysis was undertaken to evaluate the correlation between the hippocampal FC and behavioral findings.

**Results:**

Compared with the control group, the SAE group showed increased FC between the bilateral hippocampus and thalamus, septum, bed nuclei stria terminalis (BNST), left primary forelimb somatosensory cortex (S1FL), primary motor cortex (M1), and inferior colliculus. Increased FC between the left hippocampus and thalamus, septum, BNST, left S1FL, and inferior colliculus was observed. While with the right hippocampus, FC in thalamus, septum, left S1FL and inferior colliculus was enhanced. Additionally, positive correlations were found between the hippocampal FC and the immobility time in the forced swimming test.

**Conclusion:**

Hippocampus-related brain networks have significant alterations in rats with SAE, and the elevated hippocampal resting-state FC was positively related to affective deficits. Changes in FC between the hippocampus and other brain regions could be a potential neuroimaging biomarker of cognitive or mental disorders triggered by SAE.

## Introduction

Around 70% of patients with sepsis develop sepsis-associated encephalopathy (SAE), a generalized malfunction of the brain caused by infections that occur outside of the central nervous system (Andonegui et al., 2018; Chen et al., 2020). Patients with SAE usually manifest as severe and long-term cognitive impairment in the chronic phase (Semmler et al., 2013). In addition to cognitive damage, survivors also have a higher rate of psychiatric illnesses, such as depression, anxiety, post-traumatic stress disorder, and a proclivity to self-harm, than the general population. Long-term cognitive deficits and negative emotions provoked by SAE are clearly detrimental to the patients’ quality of life. However, the pathophysiology of SAE is intricate and the mechanisms causing neurological dysfunction are still inconclusive. Relevant studies have shown that SAE may be related to neuroinflammation, impaired vascular function, neuroendocrine dysfunction, neurotransmission disturbances, and other mechanisms (Sharshar et al., 2003; Semmler et al., 2007; Goodson et al., 2018). These aberrant processes are especially susceptible to specific brain regions involved in arousal, autonomic regulation, determining behavioral response to stress, and more sophisticated cognitive tasks. Therefore, the pathological alterations in or between these brain regions and corresponding behavioral changes in SAE patients have always been hot and difficult issues in clinical research.

Resting-state functional magnetic resonance imaging (rs-fMRI) is a promising application for exploring brain networks, which has been widely employed in both healthy and pathological states to research the brain and its functional structure (Lee et al., 2010; Hutchison et al., 2012; Lei et al., 2014). The resting-state functional connectivity (FC) measures the temporal dependency between neuronal activity patterns across anatomically distinct brain regions, and it sheds new light on how functional brain networks relate to human behavior (Qiu et al., 2016). A similar method can be used in rats to acquire translational measurements in models of neuropsychiatric disorders. Previous imaging studies demonstrate that the FC within the default-mode network (DMN) was significantly increased in rats with SAE, which promoted the pathophysiological understanding of cognitive and affective impairment in SAE from the perspective of neuroimaging (Ji et al., 2018).

The hippocampus is known to exist as the primary site of nerve formation in the adult brain, which participates in mediating cognition and emotion (Jinno, 2011). There is some evidence to suggest that abnormal hippocampal neurogenesis is an essential pathogenic trait of SAE (Yin et al., 2020). Furthermore, molecular targets are modified in animal models of sepsis, which have a predominant impact on the hippocampus (Comim et al., 2010; Steckert et al., 2017). Pathological changes in the hippocampus caused by SAE may lay the groundwork for the occurrence of cognitive dysfunction and affective disorders. Nonetheless, it remains unknown how the hippocampus interacts with other related brain regions, as well as its relationship with impairment of cognitive or emotional functions in individuals with SAE. Considering the pronounced vulnerability of hippocampal functional organization to the complex neuropathological processes of sepsis-related injury, we picked the hippocampus as the region of interest (ROI) and assumed that the hippocampal FC would be altered in SAE rats.

The objective of this research is to investigate the features of hippocampus-related brain networks in rats with SAE, and to further explore the relationships between the altered FC and cognitive and affective disorders. Our findings may provide a new key reference for comprehension of the neural mechanisms underlying SAE.

## Materials and Methods

### Animals and Modeling

The experimental procedures were approved by the Ethics Committee of the Second Affiliated Hospital of Nanjing Medical University and carried out in compliance with the guidelines for Care and Use of Laboratory Animals in China. Thirty male Sprague-Dawley rats (300–380 g) obtained from Nanjing Medical University were housed in a temperature-, light/dark-controlled animal care facility with unlimited access to chow and water. All rats were assigned to control and SAE groups at random (*n* = 15 per group). The rats in the SAE group were given a single dose of 5 mg/kg lipopolysaccharide (LPS) intraperitoneally. Controls were administered intraperitoneally with an equal volume of normal saline. To avoid circadian variations, all the injections were performed between 9:00 a.m. and 10:00 a.m.

### Behavioral Studies

The open field test (OFT) and forced swimming test (FST) were conducted to evaluate cognitive function and affective behaviors in the rats after 48 h of modeling. Rats were randomly divided into the SAE group and the control group to counterbalance potential order effects. Each rat was evaluated independently, with no other rats’ performance in the next tasks being seen. There was an interval of 1 day between tests to reduce the possible impact of prior testing.

#### Open Field Test

A dark gray plastic box (60 × 60 × 40 cm^3^) was utilized as the apparatus. Each test lasted 5 min and each rat was positioned in the reaction chamber’s central area. Ethovision software (version 11.5) was used to track and quantify the total distance traveled and center time. The exploration time of rats in the center zone which is 25 × 25 cm square, was calculated as the center time. The device will be cleaned before the next test.

#### Forced Swimming Test

We used an 80-cm-high transparent glass circular cylinder with a 40-cm inner circumference. The water in the cylinder was kept at a height of 30 cm and a temperature in the range of 23–25^°^C. It was difficult to modify the effects of the forced swim through behavioral adaptation since they couldn’t simply stand at the bottom of the container at a depth of 30 cm. Each test lasted 6 min after the rats were individually placed. In the remaining 4 min, the rat’s immobility time was analyzed and documented by the monitoring system. The water will be replaced before the next test.

### Magnetic Resonance Imaging Acquisition

All MR images were acquired with a 7.0 T MRI scanner (Bruker-Biospin, Ettlingen, Germany) with a four-channel phase array rat head coil after 24 h of modeling. 5% isoflurane in oxygen and air was used to produce anesthesia, and dexmedetomidine (0.015 mg/kg) was injected subcutaneously. The rats were then placed in a prone posture on a small animal MRI scan bed and their heads were fixed with dental hooks and ear bars to reduce head movements. We employed an anesthetic regimen that included a subcutaneous delivery of dexmedetomidine (0.03 mg/kg) followed by inhalational isoflurane for anesthesia maintenance. Throughout the scanning period, the degree of anesthesia was maintained by modulating the isoflurane concentration (0.2–0.5%) to preserve the respiration rate of 80–100 breaths per minute and oxygen saturation of 95–100%. Magnetic resonance scanning was started after the respiration, heart rate and saturation of rats were observed to be stable. Functional images were obtained using a single-shot gradient-echo EPI sequence (TR = 2,000 ms, TE = 19 ms, FOV = 3.2 × 3.2 cm^2^, matrix size = 64 × 64, slice thickness = 1.0 mm, slice gap = 0, voxel size = 0.5 × 0.5 × 1 mm^3^, repetition = 180).

### Image Processing and Data Analysis

Raw rat EPI datasets were firstly converted to 32-bit NIFTI format using Bruker2Analyze Converter and MRIcron.^1^ The voxel size of rat images was scaled by ten times (5 mm × 5 mm × 10 mm) to facilitate processing. Preprocessing of the rs-fMRI data were performed using Statistical Parametric Mapping (SPM 12). The first 10 volumes of functional data were eliminated for signal equilibrium. The remaining volumes were adjusted for slice timing correction, head movement correction, and spatial normalization to a standard rat brain template (resampled to 3 mm × 3 mm × 3 mm) and smoothed with kernel of full width at half maximum (FWHM) of 4 mm. The normalization method was the same as our previous study (Lu et al., 2021). Exclusion criteria were defined at 0.1 mm and 1 degree in maximum head movement. Further analysis includes band-pass filtering, seed region selection, and calculation of FC maps. The time courses of all voxels were subjected to band-pass filtering (0.01–0.1 Hz). Based on a standard rat brain atlas of Paxinos (Valdes-Hernandez et al., 2011), seed ROIs were chosen in the bilateral hippocampus, left hippocampus, and right hippocampus, respectively. Pearson’s correlation coefficients were determined between the mean time course of every seed region and other brain voxels. A Fisher’s r to z transformation was used to convert whole-brain FC maps into normally distributed data.

One sample *t*-tests of zFC (fisher’s z transformed) images in the FC analysis were performed to show brain areas significantly connected to seed ROIs. Then two-sample *t*-tests of zFC images were performed between the SAE group and control group within the masks generated by the union of statistically significant areas in the one-sample *t*-tests of the two groups. A Gaussian Random Field correction method with a voxel defining threshold *P* = 0.001, corresponding to a cluster level of *P* = 0.05 (two tails) was used for the multi-comparison correction. Data analysis was performed using SPSS for Windows software (version 26.0). Results are presented as mean ± SD. The Kolmogorov-Smirnov test was applied to ensure normal distribution of all data. Data were analyzed with two-sample *t*-tests. Pearson’s correlations were employed to assess the relationship between the FC values and behavioral performance. Statistical significance was defined as a *P*-value of less than 0.05. FDR (*q* = 0.05) was used to rectify multiple comparisons in correlation analysis.

## Results

### Behavioral Test Results

**FIGURE 1:**
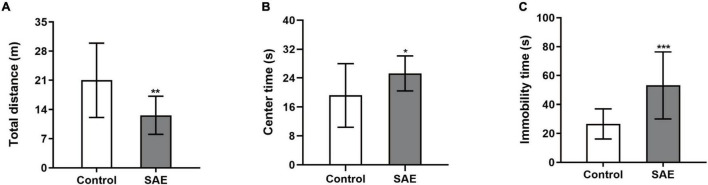
**(A–C)** Rats with SAE showed a decreased total distance and increased center time in the open field test while they displayed prolonged immobility time in the forced swimming test. *P < 0.05, ** *P* < 0.01, ***P < 0.001.

In the open field test, rats with SAE had a shorter total distance (*P* < 0.01, Figure 1A) and longer center time (*P* < 0.05, Figure 1B) compared with the control group. The immobility time of the SAE group was significantly prolonged in the forced swimming test (*P* < 0.001, Figure 1C).

### Results of Seed-Based FC Analysis

**FIGURE 2:**
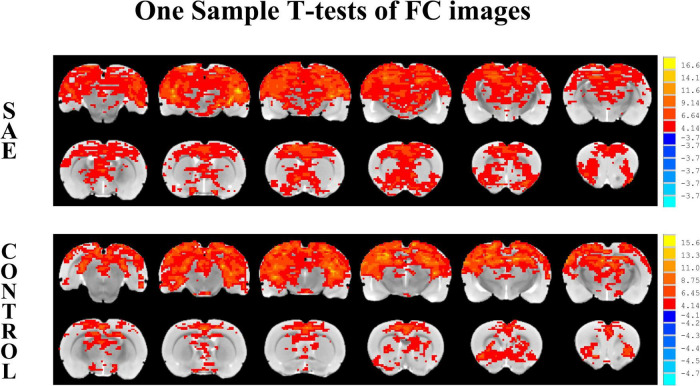
Illustration of one-sample *t*-tests results of the hippocampus functional connectivity (FC) maps in the SAE and control groups, the seed is the bilateral hippocampus.

**FIGURE 3:**
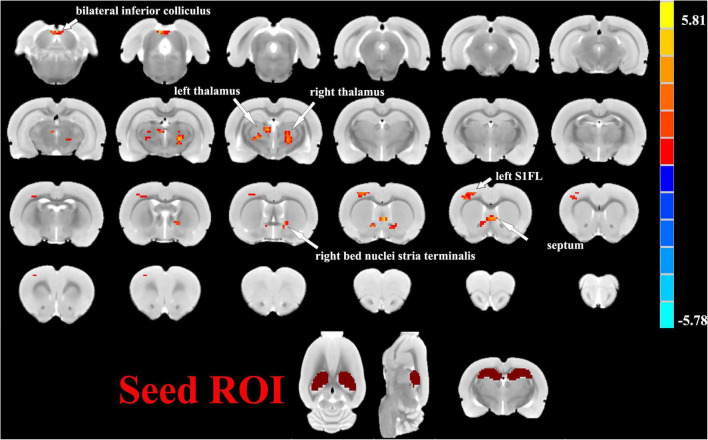
Two sample *t*-test functional connectivity (FC) analysis selecting the bilateral hippocampus as seed between the SAE and control groups. Compared to the control group, FC was higher between the bilateral hippocampus and thalamus, septum, bed nuclei stria terminalis (BNST), left primary forelimb somatosensory cortex (S1FL), primary motor cortex (M1), and inferior colliculus in the SAE group.

**FIGURE 4:**
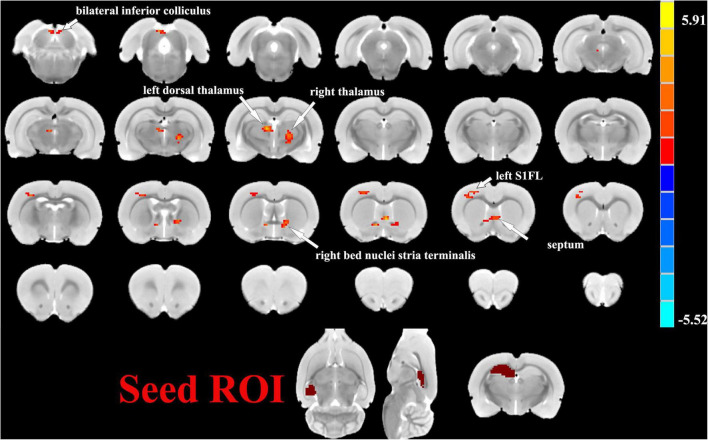
Two sample *t*-test functional connectivity (FC) analysis selecting the left hippocampus as seed between the SAE and control groups. Within the SAE group, FC analysis selecting the left hippocampus as the seed point showed significantly increased FC in thalamus, septum, bed nuclei stria terminalis (BNST), left primary forelimb somatosensory cortex (S1FL) and inferior colliculus.

**FIGURE 5:**
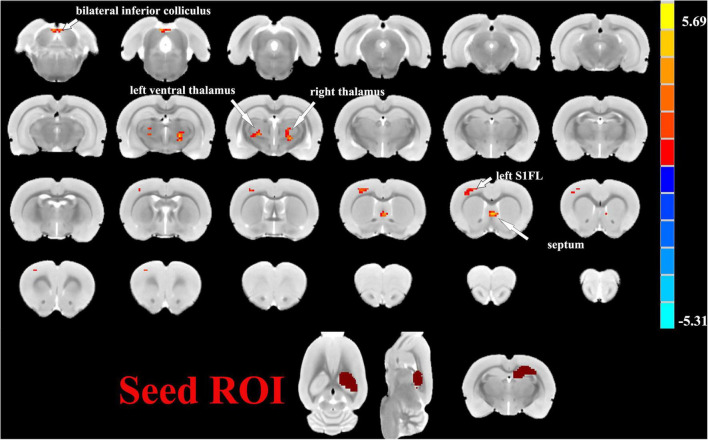
Two sample *t*-test functional connectivity (FC) analysis selecting the right hippocampus as seed between the SAE and control groups. With the right hippocampus as the seed brain region, the SAE group displayed enhanced FC between the right hippocampus and thalamus, septum, left primary forelimb somatosensory cortex (S1FL), and inferior colliculus.

One sample *t*-tests images showed brain regions significantly connected to the hippocampus (Figure 2). The pattern of the FC map connected to the hippocampus resembles the pattern of the DMN demonstrated in our previous and other researchers’ studies (Lu et al., 2012, 2021). This result verified that the hippocampus is one of the nodes in the DMN. Compared to the control group, the SAE group has higher FC between the bilateral hippocampus and thalamus, septum, bed nuclei stria terminalis (BNST), left primary forelimb somatosensory cortex (S1FL), primary motor cortex (M1), and inferior colliculus (Figure 3). FC analysis selecting the left hippocampus as the seed point showed significantly increased FC in thalamus, septum, BNST, left S1FL, and inferior colliculus in the SAE group (Figure 4). With the right hippocampus as the seed brain region, we found that the FC between the thalamus, septum, left S1FL and inferior colliculus was enhanced in the SAE group (Figure 5). Table 1 showed coordinates (anterior commissure serve as origin) of peak significance from higher FC selecting the hippocampus as the seed between the SAE and control groups.

**TABLE 1:** FC analysis selecting the hippocampus as the seed.

Brain region	Peak coordinate (Anterior commissure as origin, unit: 0.1 mm)			Volume (voxels)	T-peak
	*x*	*y*	*z*		
**Bilateral hippocampal FC**					
Left thalamus	27	6	–39	66	5.1308
Right thalamus	–18	3	–39	73	5.5627
Septum; left BNST	0	9	9	57	5.8070
Right BNST	–12	–6	3	34	5.1342
Left S1FL; M1	30	48	9	110	4.8684
Inferior colliculus	9	42	–81	39	5.2872
**Left hippocampal FC**					
Left thalamus	15	18	–36	51	5.2352
Right thalamus	–21	6	–39	56	5.1748
Septum; left BNST	0	6	6	51	5.3491
Right BNST	–12	–3	3	43	4.9559
Left S1FL	30	48	9	96	4.5596
Inferior colliculus	12	42	–78	30	4.8301
**Right hippocampal FC**					
Left thalamus	27	6	–39	31	5.1277
Right thalamus	–18	3	–39	58	5.5843
Septum	0	9	9	39	5.3961
Left S1FL	30	48	9	79	4.5682
Inferior colliculus	9	42	–81	35	5.2091

*For converting to PAXINOS & WATSON stereotaxic coordinate, just add y to 70 (y + 70). FC, functional connectivity; BNST, bed nuclei stria terminalis; S1FL, primary forelimb somatosensory cortex; M1, primary motor cortex.*

### Correlation Analysis

**FIGURE 6:**
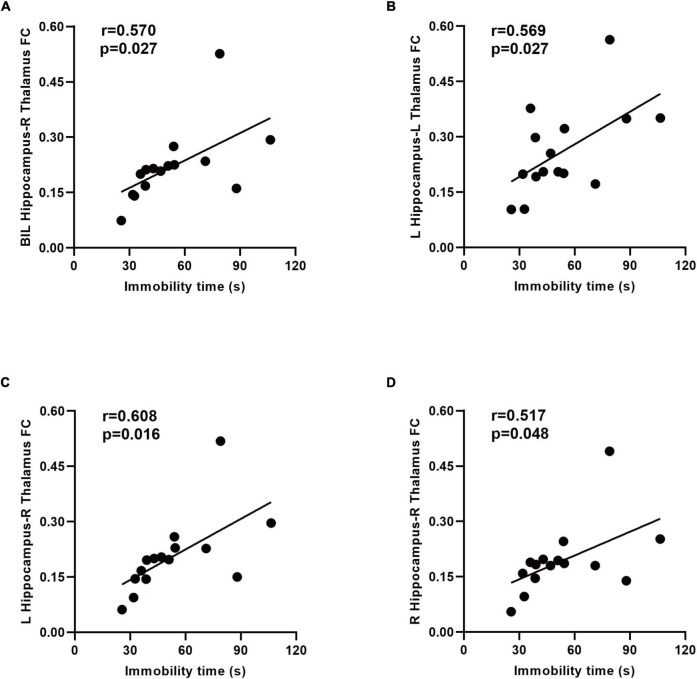
Relationship between the immobility time and functional connectivity (FC) in the SAE group. A positive correlation between the immobility time and zFC values between the bilateral hippocampus and right thalamus was found **(A)**. There was a positive correlation between the immobility time and zFC values between the left hippocampus and thalamus **(B,C)**. Increased connectivity between the right hippocampus and right thalamus associated with the immobility time in the forced swimming test **(D)**. BIL, bilateral; L, left; R, right; FC, functional connectivity.

The mean zFC values within clusters that revealed a substantial difference between the two groups were extracted. The mean zFC values between the hippocampus and thalamus is positively correlated with the immobility time in the forced swimming test (Figures 6A–D).

## Discussion

To the best of our knowledge, this is the first study to reveal the altered FC of the hippocampus in rats with SAE. Specifically, the hippocampus-related brain regions with significant differences in FC are thalamus, septum, BNST, S1FL, M1, and inferior colliculus. Moreover, mental disorders reflected by the behavioral test positively correlated with the FC of the hippocampus-thalamus.

In our study, rats with SAE exhibited some cognitive deterioration and emotional disorders such as depression and anxiety, which agreed with the findings of many clinical trials. Neuroimaging studies of the hippocampus in individuals with either depression or anxiety disturbances reveal that hippocampal structural and functional plasticity exists abnormalities. In addition, defects in hippocampal neurogenesis have been proposed to cause cognitive impairment and depression- and anxiety-like behaviors (Kim et al., 2015; Fang et al., 2020). It might explain the common incidence of cognitive dysfunction in affective disorders, as well as the depression and anxiety seen in patients with or at risk for cognitive decline. The detailed underlying mechanism remains to be further elucidated.

We discovered that the thalamus showed increased connectivity to the hippocampus in the model of SAE. As a critical structure in cognitive and mood networks, the thalamus absorbs and integrates information from the basal ganglia, limbic system, and cerebellum before relaying it to the hippocampus and the neocortex (Wolff and Vann, 2019). Injury to the thalamus which sends and receives bi-directional fibers to and from the hippocampus subiculum can induce the related networks to malfunction. The connectivity between the hippocampus and thalamus has been regarded as an important pathway for cognition (Aggleton et al., 2010). Mousavi et al. (2017) found the abnormal FC of the hippocampus-thalamus could have relevance for study into the processes driving cognitive problems in patients with absence epilepsy. Baumgartner et al. (2018) detected that cognitive deficits imposed by stroke were closely related to disruption of hippocampal-thalamic connectivity. Furthermore, a fMRI study of a highly anxious population indicated aberrant connectivity of the left hippocampus and thalamus (Yang et al., 2019). Research has demonstrated that the connectivity between the hippocampus and thalamus affects depression to a large degree (Im et al., 2021). In our study, the thalamus was shown to have enhanced connectivity with the hippocampus, and the enhanced FC values between the two regions were negatively correlated with behavioral performance in rats with SAE. Our results were consistent with previous studies showing that the destroyed connectivity of the thalamo-hippocampus may be associated with cognitive deficits and mood disorders (Yang et al., 2019). These findings were interpreted as implying the existence of neural self-compensation or cerebral reserve (Li et al., 2020). Increased FC between the hippocampus and thalamus may also result from elevated external somatosensory stimuli to the central nervous system, especially to the hippocampus or thalamic nuclei from the systemic inflammatory response in SAE rats.

Our data also showed the increased FC between the hippocampus and septum in rats with SAE. The hippocampus and the septum are at the heart of the limbic system, managing motivated behavior and regulating numerous cognitive tasks. Complex reciprocal connectivity in anatomy and function has been demonstrated between the hippocampus and septum (Okada and Okaichi, 2010). These connections form the hippocampo-septal and septo-hippocampal pathways which are linked to cognitive functions (Niewiadomska et al., 2009). In line with several study findings, the oscillations in the septo-hippocampal network are influenced by depression and engaged in its symptoms (Hajos et al., 2003; Kang et al., 2010). Anxiety-related activities are thought to be bound up with theta oscillations in the septo-hippocampal circuits (Caliskan and Stork, 2019). One possible explanation is that in our study, the enhanced FC of these regions in SAE rats is a compensatory mechanism as cognition or emotion becomes impaired. The increased resting-state hippocampal connectivity to the septum may be interpreted to mobilize additional neural resources to maintain cognitive or affective status in SAE patients.

The BNST, which is a complex cluster of neuronal nuclei situated in the basal forebrain, has been implicated in the negative affective state or psychiatric disease (Lebow and Chen, 2016). With strong connectivity to the hippocampus, the BNST is localized more specifically to the anterior CA region that is involved in anxiety reactions and motivation processing (Torrisi et al., 2015). We observed both the bilateral hippocampus and left hippocampus showed increased FC with the BNST, suggesting that it might be the potential mechanism of depression- and anxiety-like behaviors due to SAE.

Located in the postcentral gyrus, the primary somatosensory cortex (S1) is closely linked to the thalamus both structurally and functionally (Zhang et al., 2010). The S1 is regarded as a sensory signal structure for acquisition and transformation; nonetheless, its role in controlling and regulating associative learning behavior has also become increasingly evident (Galvez et al., 2006; Chau et al., 2013). (Kang et al. (2018) found that increased S1-thalamic FC in individuals with major depressive disorder was linked to cognitive function and affective experience. As shown in previous research, reduced synchronization between the hippocampus and S1, in particular, correlates with cognitive and memory impairment in aged rats (Xie et al., 2013; Ash et al., 2016). Our results add to the growing body of evidence indicating the hyper-connectivity between the two is crucial. This discovery might lead to more information that disrupted functional networks between the hippocampus and S1 underlie the defective cognitive and emotional ability of SAE patients. Since the M1 is located adjacent to the S1FL region in the rat brain, our result may be falsely positive due to technical issues such as inadequate registration or normalization. Nevertheless, the increased connectivity between the bilateral hippocampus and M1 may be interpreted as the compensatory effects after the motor or cognitive-affective function was impaired, or the connectivity changed after the activity in M1 was affected by SAE, and the specific mechanism required further study.

In addition to the findings presented above, we detected the increased connectivity between the hippocampus and inferior colliculus in the SAE group. The inferior colliculus serves as a pivotal relay station for auditory pathway (De Martino et al., 2013). Schwenzer et al. (2012) revealed that auditory impairment could be a sign of depression. Studies on elderly individuals with sensory deficits found that the damaged auditory function was linked to an increased incidence of depressed and anxious symptoms (Simning et al., 2019). Our work demonstrated alterations in FC between the hippocampus and inferior colliculus, which might be related to the disordered emotions or auditory cognitive defects seen in rats with SAE.

Our research has several limitations. First, although the spontaneous activity of rats decreases when they are depressed, it is undeniable that the increased immobility time of the SAE rats might be associated with a decrease in motor function. Second, we employed a seed-based analysis, which has an underlying methodological constraint caused by *a priori* hypothesis about the seed region for the extraction of BOLD time-courses to assess the temporal correlation with the time series of other voxels in the brain. The hippocampus could be more finely divided into different subregions according to diverse functions, however, we did not subdivide it more accurately when choosing ROIs. Lastly, our study prevented us from investigating causal relationships or the role of change in hippocampus-related brain function on the evolution of cognitive or emotional deterioration in rats with SAE. Other unmeasured variables could be responsible for the correlation between them, thus more research is needed.

In summary, the current study provides evidence that the increased activity in the hippocampus functional network likely contributes to cognitive and emotional deficits in the SAE rat model. The hippocampus-related brain function may be an effective imaging target for cognitive disturbances and affective experience in SAE. The specific altered region could provide a valuable reference for further understanding the etiology of SAE.

## Data Availability Statement

The original contributions presented in this study are included in the article/supplementary material, further inquiries can be directed to the corresponding author/s.

## Ethics Statement

The animal study was reviewed and approved by the Ethics Committee of The Second Affiliated Hospital of Nanjing Medical University.

## Author Contributions

YY and CL conceived the experiments, analyzed the data, and wrote the first draft of the manuscript. YY, JS, CZ, CT, and XX performed the experiments. JC, JT, and HY contributed to the conception and design of the study and revised the manuscript. All authors contributed to the article and approved the submitted version.

## Conflict of Interest

The authors declare that the research was conducted in the absence of any commercial or financial relationships that could be construed as a potential conflict of interest.

## Publisher’s Note

All claims expressed in this article are solely those of the authors and do not necessarily represent those of their affiliated organizations, or those of the publisher, the editors and the reviewers. Any product that may be evaluated in this article, or claim that may be made by its manufacturer, is not guaranteed or endorsed by the publisher.

## Footnotes

1
http://people.cas.sc.edu/rorden/mricro/
